# Lacrimal Sac Tumors: A Histotype-Driven Literature Review

**DOI:** 10.3390/cancers17223718

**Published:** 2025-11-20

**Authors:** Luca Giovanni Locatello, Enrico Redolfi De Zan, Riccardo Marzolino, Leigh J. Sowerby, Anna Tarantini, Paolo Lanzetta, Cesare Miani

**Affiliations:** 1 Department of Otorhinolaryngology, Academic Hospital “Santa Maria della Misericordia”, Azienda Sanitaria Universitaria Friuli Centrale, Piazzale Santa Maria della Misericordia 15, 33100 Udine, Italy; 2 Department of Medicine—Ophthalmology, University of Udine, 33100 Udine, Italy; 3 Department of Otolaryngology—Head and Neck Surgery, Western University, London, ON N6A 3K7, Canada; 4 Istituto Europeo di Microchirurgia Oculare (IEMO), 33100 Udine, Italy; 5 Department of Medicine (DMED), University of Udine, Via Colugna 50, 33100 Udine, Italy

**Keywords:** lacrimal sac cancer, dacryocystectomy, dacryocystorhinostomy, lacrimal sac tumor, transnasal endoscopic surgery, orbital surgery

## Abstract

Objectives: Because of their rarity, lacrimal sac tumors (LSTs) are challenging to diagnose and treat. We herein provide an overview of the recent literature. Methods: A scoping search of the Cochrane library, PubMed and Google Scholar database in the last 5 years was conducted. Three independent reviewers extracted data, and the findings were summarized due to study heterogeneity. Results: A total of 55 articles were included. LST histology is diverse and there is no commonly accepted staging system. Recent discoveries in their biology are offering new treatment strategies but exclusive endoscopic resections remain feasible in only very limited cases of non-aggressive LSTs. Conclusion: LSTs require a high index of suspicion because of their rarity. A histotype-driven treatment plan must be carefully prepared, but complete excision remains the cornerstone of treatment in all cases.

## Simple Summary

Lacrimal sac tumors are represented by a large array of benign and malignant histologies. Because of their rarity, diagnosis is usually delayed and the management remains unclear. Improvements in imaging and transnasal endoscopy have brought practical innovations for these lesions that need a multidisciplinary expertise from head and neck surgeons to ophthalmologists. Establishing a commonly accepted staging system is a priority for these tumors, while surgical resection also with the aid of endoscopy remains the cornerstone of treatment. Future multi institutional registries are fundamental for strengthening the evidence in this field.

## 1. Introduction

The lacrimal sac resides at the junction of the lacrimal bone and the frontal process of the maxilla, thus representing a shared space for both ophthalmologists and rhinologists [1]. This ectodermal structure is lined by a pseudostratified columnar epithelium (including cilia and goblet cells), and with a nearly constant small amount of associated lymphoid tissue. It receives tears from the two canaliculi through the valve of Rosenmuller and continues as a nasolacrimal duct after the valve of Krause [2]. The sac is the surgical target of both endoscopic and open dacryocystorhinostomy (DCR) [3,4]. These procedures are aimed at relieving distal lacrimal obstruction (DLO), which, on very rare occasions, may be caused by benign or malignant primary lacrimal sac tumors (LSTs) [5]. Epidemiologically, tumors are rare (less than 1000 cases reported worldwide), diagnosis is often delayed, staging systems are practically non-existent, and treatment is based on anecdotal reports in the available literature [5]. The scarce evidence is additionally favored by the complex histological classification of LSTs. The latter was revised in 2023 with the publication, from the World Health Organization (WHO), of the Fifth Edition of the Classification of Eye Tumors [6].

There has been a recent increase in papers regarding LSTs, but the most recent reviews on the subject are more than five years old [5,7]. While LSTs were classically treated with external dacryocystectomy (a procedure that dates back to 1724), the widespread diffusion of endoscopic techniques is rapidly changing the management of LSTs [8,9]. The aim of the present scoping review is to provide an updated report of the most recent literature about LSTs. After a general overview, new clinicopathological insights for the most common histotypes will be reported in detail, while a qualitative synthesis is given for the less common histotypes.

## 2. Materials and Methods

**Figure 1:**
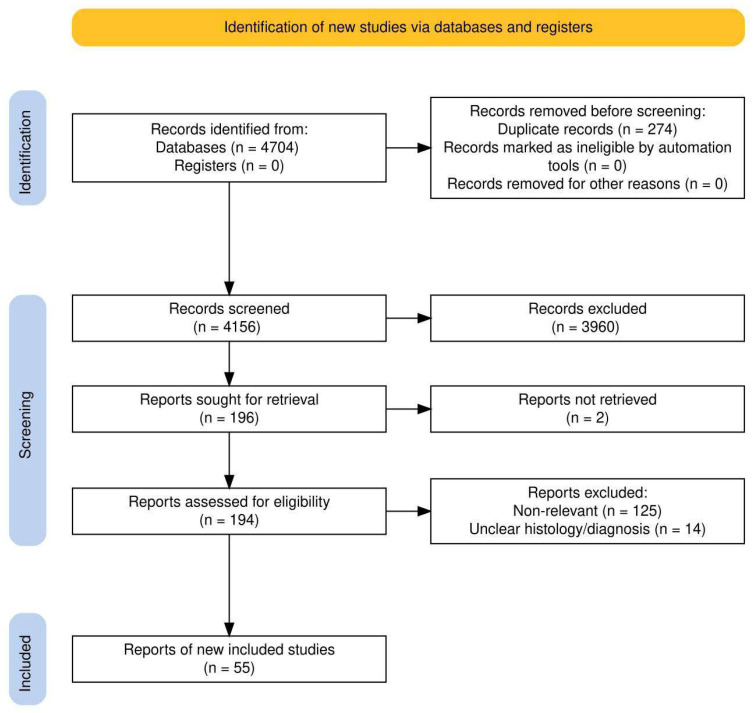
PRISMA flowchart for the selection of the studies discussed in the present paper.

The PRISMA statement was followed in the preparation of the present paper, and a modified PRISMA flowchart is given in Figure 1 while a checklist is available as a Supplementary file [10]. The flowchart was generated using the free web-based Shiny app as provided by Haddaway and coworkers [11]. No institutional review board approval was necessary for the present work. The present review was inserted into the Open Science Framework—OSF Public Registry with the registration reference DOI https://doi.org/10.17605/osf.io/hxtsu (Center for Open Science, Charlottesville, VA, USA; the whole protocol can be found at osf.io/37g5aaccessed on 14 September 2025).

PEO (Population, Exposure, and Outcome) was as follows: P (cases of LSC), E (any surgical and non-surgical treatment), and O (survival and treatment complications). The Cochrane Library, PubMed, and Google Scholar databases were used in order to perform a review of the literature from 1 January 2020 to 1 October 2025. The following search string was used: [PubMed Medline] and [Cochrane Library] “lacrimal sac AND tumor OR cancer OR neoplasms”; [Google Scholar] “lacrimal sac (tumor OR cancer OR neoplasm)”. Duplicates were removed with Zotero [12]. All the pertinent articles were included after careful reading of the titles and abstracts. Full texts of the included articles were then retrieved by three authors and quantitative and qualitative data were synthesized accordingly. Any paper describing the management of histopathologically proven lacrimal sac tumor(s) was included. Exclusion criteria are inflammatory disorders of the lacrimal sac; biopsy-unproven tumors of the lacrimal sac; mixed outcomes for tumors of the orbit/gland/sac; non-relevant or off-topic papers; book chapters, conference presentations or conference papers; and studies written in languages other than English, French, or Italian.

The search strategy retrieved a total of 4156 articles and, after applying the selection criteria and checking through the reference lists of the relevant studies, a total of 55 full texts were ultimately analyzed (Figure 1). Five further studies were also added during the revisions of the manuscript. Quantitative and qualitative data regarding surgical outcomes were summarized and are systematically reported in tables.

## 3. Results

### 3.1. Clinical Presentation and Diagnostic Work-Up

A general classification of primary LSTs is given in Table 1. Historically, epithelial tumors have been considered the most frequent histotype, but in the latest series, lymphomas seem most prevalent [13,14,15]. Given their rarity, there is no clear risk factor or gender preference for LSTs. A molecular characterization of their genetic landscape has been recently presented with salivary-derived cancers showing TP53 and CIC mutations and amplification of ERBB2, while transitional cell carcinoma seemed associated with HPV-16 [16].

These neoplasms usually appear in the middle age. In children, congenital dacryocystocele is the principal cause of medial canthus swelling. Clinical presentation overlaps with inflammatory DLO. In particular, tears mixed with blood (i.e., hemolacria) is reported in 0–40% of LST cases but it is associated with malignancy only in 8% of cases [17,18]. Diagnostic delay is common: in two Asian series, it was 14.7 (median 8; range 1–96) and 22.4 months (median 10; range 2–120) [15,17]. In another series of 65 LSTs (but where half of them were secondary malignancies), patients with benign lesions were older than those with malignancies [19].

Laboratory tests are of little value in the work-up of LSTs. Even in lymphoid neoplasms presenting as a pseudo-dacryocystitis, the underlying hematological issue is already known in 88% [20]. LST imaging is otherwise crucial; contrast-enhanced computed tomography (CECT) and magnetic resonance imaging (MRI) with the use of small surface coils are the workhorses for all lacrimal disorders, including LSTs. While local extension is accurately appreciated with current protocols, there are no clear-cut patterns that can help in distinguishing benign from malignant lesions, except for obvious advanced-stage lesions. Secondary involvement of the lacrimal sac may occur from tumors originating from adjacent structures, and in the differential diagnosis, tumor-like lesions in the context of a systemic illness (sarcoidosis, granulomatosis with polyangiitis, IgG4-related disease) must also be considered. Finally, radiological signs such as erosion/remodeling of the lamina papyracea or the nasal bone, as well as cutaneous fistulas are nonspecific and can sometimes be present even in chronic dacryocystitis. Biopsy is thus essential for diagnosis [21]. Lastly, a source of heterogeneity in reporting malignant LSTs is the lack of a commonly accepted staging system analogous to the AJCC Tumor, Node, and Metastasis (TNM) classification.

### 3.2. Principles of Treatment for LSTs

External dacryocystectomy, usually through a modified Weber–Ferguson incision, remains the gold standard approach for all resectable LSTs [8]. Historically, an R0 (negative) surgical extirpation of the lesion was the cornerstone of treatment, but this would occasionally require the sacrifice of the adjacent skin, bone, and orbital contents. Adjuvant treatments in the form of external radiation therapy (RT) or chemotherapy were reserved for R1 (microscopically) or R2 (macroscopically positive margins) cases, or in case of more aggressive histotypes [13,14,15]. More recently, combined or even solely transnasal approaches have been brought forward: a pooled analysis of 316 LST cases published in the years 2013–2023 found that 58.9% had exclusively open resection, 7.9% had combined open and endoscopic, while only 1.9% were treated exclusively by a transnasal approach. Interestingly, endoscopic transnasal biopsy was feasible in 73.9% of patients [9]. Endoscopic assistance, also with the aid of an image-guided navigation system [22], remains important in transfacial resection: a recent series from Shanghai achieved a 84.6% (11/13) disease-free survival with a mean follow-up time of 58.6 months [23]. Of note, the first robotic-assisted globe-sparing resection of a pT1 squamous cell carcinoma of the lacrimal sac was reported in 2024 [24].

Cervical nodal status is prognostically important: in a recent series from Philadelphia, 40% of malignant LSTs were considered cN+ and 60% of them were pN+ at final histopathological report. Unfortunately, the authors do not report the histotypes but they recommend that neck dissection (again, levels are not reported) and ipsilateral parotidectomy to be performed in “high-risk” tumors [25].

While adjuvant RT is commonly administered, radical RT is not because it is associated with visual dysfunction, cataract, glaucoma, and radiation-induced neuropathy. In this regard, intensity modulated proton therapy (IMPT) has been successfully tried in one case of LST SCC [26]. An alternative is offered by brachytherapy, and in 2020 the first experience was published in a Chinese report [27]. After orbit-sparing surgery, 4 patients (1 SCC, 1 ACC, 1 lymphoma, and 1 adenocarcinoma) had I-125 seeds left into the nasolacrimal canal or under the orbital periosteum; with a median follow-up time of 28 months, all patients were alive, with satisfactory visual function, and with only one case of dermatitis as a reported side effect [27].

Outside of treating metastatic disease, chemotherapy has been used in neoadjuvant settings with the aim of avoiding orbital exenteration. In a case series of seven patients with LS carcinomas from the MD Anderson Cancer Center, a combination of several drugs (platinum-based, taxane, and cetuximab or pembrolizumab) demonstrated good oncological outcomes [28]. There is a 2020 Chinese case report of a bulky SCC where, in an effort to preserve the globe, transcatheter arterial infusion of lobaplatin and docetaxel was given along with embolization of polyethylene microspheres, followed by IMRT. While the shrinkage of the mass was remarkable, the patient was lost to follow up and this approach remains highly experimental [29].

### 3.3. Clinicopathological Update on the Most Common LST Histologies

A histotype-driven description of the most commonly encountered LSTs is given in the following sections. For the sake of completeness, all the papers discussing very rare histologies of primary LSTs are briefly presented in Table 2 [30,31,32,33,34,35,36,37,38,39].

### 3.4. Inverted Papilloma

Inverted papilloma (IP) is a benign epithelial neoplasm known for its peculiar biological behavior, demonstrating local aggressiveness, a significant tendency for postsurgical recurrence, and an inherent potential for malignant transformation [40,41]. Primary IP of the LS is considered extremely rare. The etiology of IP is not completely understood, but it is hypothesized that it may arise from metaplasia of the Schneiderian epithelium in response to chronic inflammation, and in some series the term “transitional cell tumor” is used. As any other primary LSTs, signs and symptoms are nonspecific and nasal endoscopy may only reveal a bulge on the lateral nasal wall corresponding to the projection of the lacrimal sac [40,41]. CT scan typically shows a soft-tissue density mass located in the LS, and signs of bone remodeling and partial dehiscence of the lamina papyracea are frequently observed. A peculiar feature, classically described for sinonasal IPs, is the “convoluted cerebriform pattern,” visible especially on STIR or T2-weighted sequences on MRI, and this correlates with the tumor’s endophytic (inverted) growth pattern. Ito et al. also showed that IP demonstrated a marked FDG uptake (SUV_max_ of 7.34) by [18F] fluorodeoxyglucose Positron Emission Tomography/Computed Tomography (FDG-PET/CT). This finding is significant because such value is usually associated with coexistent malignancy, which was not confirmed at final histopathology [40]. Endoscopic resection was initially attempted in the case by Ito et al., but despite negative intraoperative frozen sections, a recurrence was noted after one year [40]; in the other case, combined resection (endoscopic maxillectomy + lateral rhinotomy) with negative resection margins was again unsuccessful since recurrent disease appeared after few months [41]. Analogous to sinonasal IP, addressing the pedicle of origin is mandatory but anatomical reasons make this concept much more challenging; therefore, all the authors suggest frequent endoscopic evaluation in the first year (e.g., every 3 months), followed by checks every 3–6 months up to the fifth year, and subsequently annual long-term consultation [40,41].

### 3.5. Squamous Cell Carcinoma

SCC is one of the most frequent LSTs, around 70–81% of cases, including the 15–20% forms of transitional cell carcinoma (TCC, a non-keratinizing SCC). SCCs are followed in frequency by mucoepidermoid carcinoma (MEC) at 7–8%, adenocarcinoma at 6%, and adenoid cystic carcinoma (ACC) at 4% [5,42]. These malignancies are characterized by a high likelihood of recurrence (reported rates vary from 11% to 66%), and significant mortality, potentially ranging from 10% to 40%, although overall mortality in one review was 15% [36,42,43,44]. Cases of pediatric and metachronous bilateral SCC have been documented [45]. Risk factors include pre-existing papillomas and chronic dacryocystitis, although this may be a misunderstanding of the basis of prior symptoms [42]. Viral infection may also play a role: Epstein–Barr virus (EBV) was especially linked to lymphoepithelial carcinoma [42,46]. A recent report showed that human papillomavirus (HPV) is associated to both papillomas (low-risk serotypes 6/11) and epidermoid carcinomas (p16+, high-risk serotypes 16/18) [47]. Another study reported HR-HPV positivity in 6/7 LS SCCs analyzed, while older reviews suggest HPV positivity in 67–89% of investigated SCCs [5,45]. HPV association has been linked to younger age, transitional histology, p16 positivity, and loss of Rb expression [45,47]. Smoking is also cited as a potential risk factor [46]. Typical SCC markers include cytokeratins (CK5/6), p63, and p40. p16 positivity can indicate HPV infection and potentially greater chemosensitivity [45], while p53 expression is also reported [42]. Tumors often show stable microsatellite instability (MSI) and low tumor mutational burden (TMB). PD-L1 overexpression suggests potential benefit from PD-1/PD-L1 inhibitors, although hyper-progression has been observed [45,46]. There is no accepted TNM staging system for LSTs. A proposed unvalidated one [42] is

Stage I Confined to LS fossa;
Stage II Invasion of globe, nasolacrimal duct, canaliculi, caruncle, or conjunctiva;
Stage III Invasion of nasal cavity/paranasal sinuses, bone, or skin;
Stage IV Extension to orbital apex, meninges, brain, N+, or M+.

Advanced local extension may require more extensive sinus resection, lateral rhinotomy, or orbital exenteration. The latter was required in 18% of cases in older series, but its impact on survival compared to eye-sparing surgery is debated [5,44]. Besides surgery and adjuvant RT, new emerging strategies include Tyrosine Kinase Inhibitors (Anlotinib) and anti-PD-1 antibodies (Cemiplimab). Cemiplimab combined with cisplatin-docetaxel showed excellent response in one advanced SCC case [48]. Overall, 5-year overall survival ranges from 61% to 88% [44,45,46,47,48]. Orbital SCC carries a worse prognosis than conjunctival or LS SCC [49, 50]. Nodal metastases (reported in ~17–27% historically) are a key negative prognostic factor associated with worse survival and higher recurrence [42]. Metastases can occur early, even after R0 resection [49]. Poor prognostic factors include advanced stage, nodal involvement, R1, and specific molecular alterations [44,45,46,47,48,49].

### 3.6. Lymphoproliferative Tumors

Lymphoproliferative tumors of the lacrimal sac are among more common LSTs [51,52]. Among 63 primary cases, diffuse large B-cell lymphoma (DLBCL, 43%) and mucosa-associated lymphoid tissue (MALT) lymphoma (24%) were the most common variants, with mean age at presentation of 50 years among the 102 cases of primary and secondary lymphomas and leukemias [51]. Management is based on the specific lymphoma subtype and its extent. If localized, such as a MALT lymphoma, irradiation (and possibly resection) is indicated. Prognosis was poorest in patients with aggressive subtypes, particularly NK/T-cell lymphoma [51]. Another retrospective study of 15 patients from China identified 8 cases of DLBCL, 5 of MALT lymphoma, and 2 of NK/T-cell lymphoma. All patients underwent tumor excision with or without DCR, and 12 received subsequent chemotherapy tailored to the specific lymphoma and stage. None who had a DCR developed subsequent epiphora, whereas those without DCR experienced variable tearing [53]. Ucgul described two children with MALT lymphoma found during DCR: one received subsequent chemotherapy and 30 Gy regional irradiation following excision, and the other solely irradiation. Both showed an initial complete response, but recurred one year and two years later [54]. DLBCL can show aggressive behavior with infiltration of the maxillary sinus and the infraorbital nerve, mimicking SCC [55]. Finally, a case of bilateral primary extranodal marginal zone B-cell lymphoma limited to the lacrimal sacs was reported in a 66-year-old individual treated with bilateral endoscopic DCR (including revision on one side) and six cycles of chlorambucil with prednisolone [56].

### 3.7. Mucoepidermoid Carcinoma (MEC) and Other Salivary Gland-Type Carcinomas of the Lacrimal Drainage System

Only a limited number of cases of MEC of the lacrimal sac have been reported in the literature. Gervasio et al. conducted a study involving six cases, where after radical resection, all were free of disease (mean follow-up of 18 months); they also found Mastermind-Like Transcriptional Coactivator 2 (MAML2) gene fusions in the MECs of the LS compared to MECs of the lacrimal gland. They also identified EGFR amplification in one tumor, suggesting a potential role for anti-EGFR therapies [57]. Similar alterations were also identified by Sun et al., and therefore, MECs of the LS may have a molecular profile quite different from MECs arising in other subsites [58]. Apart from MECs and other salivary benign tumors, other primary salivary gland-type carcinomas of the LS have been described: Zhang et al. reported the case of a patient with adenoid cystic carcinoma (ACC) of the lacrimal sac, who declined surgical resection and opted for combined apatinib- and nedaplatin-based concurrent chemoradiotherapy. A complete response was observed just three weeks after treatment, and the patient remained disease-free for 22 months post-treatment [59]. Another patient with LS ACC was NED at 6 years after LS resection and irradiation [60].

Another histotype was a case of rapidly growing sebaceous carcinoma of the right lacrimal sac in a 29-year-old female adult; despite the presence of nodal metastases the patient was lost at follow-up [61].

Adenocarcinoma NOS (Not Otherwise Specified) is known for the chance of exploiting androgen deprivation therapy in addition to surgery and RT. Takizawa et al. reported the case of a 72-year-old woman diagnosed with primary adenocarcinoma of the LS, which had invaded the ethmoid bone. The patient declined surgery and received exclusive RT. Five years after treatment, there was no evidence of recurrence. Despite the positive outcome in terms of tumor control, the patient developed radiation-related complications, including grade-3 retinopathy and secondary neovascular glaucoma [62]. In another paper, Abramson et al. reported a case of an 82-year-old male patient with primary LS adenocarcinoma, who opted for androgen deprivation monotherapy instead of orbital exenteration and radiation. After 4 years of treatment, and with partial local regression observed on MRI, there was no progression of the disease [63].

### 3.8. Melanoma Involving the Lacrimal Drainage System

Melanoma of the lacrimal drainage system is an extremely rare condition, with fewer than 100 reported cases in the literature. Recently, two cases from the Mayo Clinic were described where only aggressive surgery was successful in achieving no evidence of disease after four years of follow-up for a 1 × 1 cm melanoma of the LST; in the second case, where melanoma was accidentally discovered after DCR, subsequent oncologic resection along with proton therapy and immunotherapy could not avoid rapid progression of disease [64]. In another paper, Shao et al. describe the CT and MRI findings of primary lacrimal sac melanoma, typically reporting hyperintensity on T1-weighted images and hypointensity on T2-weighted images due to the paramagnetic properties of melanin; however, in contrast to previous reports, most cases do not exhibit these characteristic signals [65]. Orgi et al. also reported a case which involves distant metastases [66].

### 3.9. Metastatic Tumors to the Lacrimal Sac

Metastatic tumors to the lacrimal sac from distant sites have been reported all over the world [67,68,69,70]. In most cases, primary cancer is already known at the time of presentation and a thorough medical history pinpoints the diagnosis. A 61-year-old woman with stage IV non-small cell lung cancer presenting with left facial pain and epiphora, and a biopsy of an LS mass showed a metastasis [67]. Another reported an 8-year-old boy with refractory acute lymphoblastic leukemia who had a painless enlargement of the lacrimal sac, and biopsy was consistent with B-lymphoblastic leukemia; after chemoradiation, contralateral sac involvement occurred as well as concurrent medullary relapse [68]. A third case reported that a 41-year-old woman with stage IV breast cancer had epiphora, bloody tears, and left eyelid swelling. Biopsy showed malignant phyllodes tumor [69]. In other cases, small nodules below the medial canthus and involving the sac were confirmed as in-transit metastases from a visible eyelid melanoma [70].

## 4. Discussion

This paper updates a literature overview of LSTs, where minimal progress has been made in recent years [71] because of the many histotypes seen among the small number of reported cases. A potential confounder in older series is where the origin from the lacrimal pathway is difficult to prove. This fact, along with the many histotypes described, prevents a clear-cut evaluation of prognostic factors. Since the increasing worldwide diffusion of CT or MRI scans, it is likely in the future that a larger number of LST cases will be identified. Defining a cross-validated staging system is therefore a priority for these tumors. The aforementioned proposal of a four-tiered system (I, LS; II, globe; III, bone/skin; IV, brain/nodes/distant sites) [42] is not validated, nor does it parallel the current TNM staging system for nasal tumors. However, staging systems are meant to stratify patients by survival outcomes which, as we have outlined in the present paper, depend also on the specific LST histotype. Therefore, we feel that this classification may be viewed in terms of localized disease (stage I, and the only case potentially amenable by exclusively endoscopic resection); extended disease (stages II–III), where open transfacial resections with negative margins are the cornerstone for the majority of histologies; and far-advanced cases (IV) where surgery may not be feasible.

In the future, a histology-driven approach is conceivable for lacrimal sac malignancies, in parallel to what is currently being achieved for sinonasal malignancies [72]. At present, the chance of obtaining a minimally invasive endoscopic transnasal biopsy of any possible LST might represent the most practical advance for our patients. As LSTs are rare, establishing a global shared dataset would contribute to providing better evidence for different management perspectives.

## 5. Conclusions

Neoplasms of the lacrimal sac require a high level of suspicion because of the many conditions that may present with similar symptoms. Their rarity currently impedes a histology-driven strategy. There have been improvements in diagnosis in recent years due to both better imaging and the possibility of transnasal biopsy. Some highly selected cases of LSTs may be treated by an exclusive endoscopic approach.

## Figures and Tables

**Table 1:** The table highlights the most common tumoral and tumor-like lesions involving the lacrimal sac. Partially adapted from the latest WHO 2022 Tumors of the Eyes Classification, according to Milman et al., 2023 [6].

Benign and premalignant epithelial tumors
*Squamous Papilloma*
*Inverted Papilloma*
Malignant epithelial tumors
*Squamous cell carcinoma*
*Transitional Cell Carcinoma*
*Lymphoepithelial carcinoma*
*Mucoepidermoid Carcinoma*
*Other salivary gland-type carcinomas*
*Adenocarcinoma NOS*
Melanocytic tumors (*melanoma*)
Hematolymphoid Tumors (*e.g., B-cell Lymphoma*)
Secondary Tumors
*Extension from nearby tumors of skin, sinonasal cavities, ocular adnexa*
*Metastases from distant sites*
Tumor-like lesions
*Sarcoidosis*
*Granulomatosis with polyangiitis*
*IgG4 related disease*

**Table 2:** A clinicopathological overview of the less frequent histotypes of primary lacrimal sac tumors in the last 5 years. LST, lacrimal sac tumor; RT, radiation therapy; CHT, chemotherapy; ESS, endoscopic sinus surgery [30,31,32,33,34,35,36,37,38,39].

Author, Year	Histotype	Histopathological Examination (HPE)	Immunocytochemistry (IHC)	Treatment and Outcome
Panda et al., 2022 [30]	**Solitary fibrous tumor**	Capsulated tumor with cellular neoplasm composed of spindloid to oval cells with mild degree of nuclear atypia arranged in sheets and fascicular pattern.	Cytoplasmic and membranous positivity for CD34, CD99, STAT-6, BCL-2 and weak to moderate positivity for smooth muscle actin (SMA). KI67 was 10%	- DCR with insertion of Crawford tube + en-bloc excision - 3 years disease free
Samaddar et al., 2021 [31]	**Solitary fibrous tumor**	FNAC *: Cohesive fragments and dispersed cells with pseudoacinar arrangement and interspersed slender branching vascular channels. Tumor cells are round, ovoid, and spindled with scant wispy cytoplasm and minimal nuclear atypia, stripped nuclei and scant collagenous stroma.	Nuclear reactivity for STAT6	- Not known - * First case reported in the literature: Diagnosis with fine needle aspiration cytology (FNAC)
Sharma et al., 2020 [32]	**Epithelial–Myoepithelial Carcinoma**	2-layered epithelium arranged in interconnecting nests, tubules, and gland-like structures. The inner layer was characterized by abundant eosinophilic granular cytoplasm. The outer layer had less cytoplasm. The nuclei showed mild enlargement and pleomorphism.	The outer layer of myoepithelial cells was positive for smooth muscle actin (SMA), calponin, p63, and keratin 5/6. The inner layer of epithelial cells stained for keratin 7.	- Right maxillary antrostomy, partial ethmoidectomy, and biopsy of the nasolacrimal mass. - First reported case originating from the lacrimal sac
Almutairi et al., 2022 [33]	**Oncocytoma**	Epithelial cells arranged in an adenomatous fashion and in cords. Epithelial cells lining the acini were relatively benign and moderately eosinophilic. No mitotic figures or atypia.	CK18 positive in inner cells and P63 positive in outer cells.	- External dacryocystectomy (status of margins not stated) - 3 years later, local recurrence
Gupta et al., 2024 [34]	**Primary anaplastic extramedullary plasmacytoma**	Sheets of atypical plasma cells with eccentrically placed nuclei, with moderate nuclear polymorphism and prominent nucleoli	Membranous positivity for CD38, nuclear positivity for MUM1, and negativity for cytokeratin EBER-ISH.	- Dacryocystectomy - 10 months disease free
Kumar et al., 2021 [35]	**Angiofibroma**	Tissue lined by pseudostratified columnar ciliated epithelium with focal area of squamous metaplasia. Underlying subepithelial tissue shows blood vessels with dense lymphoplasmacytic infiltrate. The vessels are small and capillary sized lined with plump epithelial cells and surrounded by delicate fibrocellular stroma.	CD34 and SMA highlight the blood vessels and capillaries. KI67 < 2%.	- Flapless DCR - 6 months disease free
Miller et al., 2020 [36]	**Transitional cell carcinoma**	Papillomatous lesion with both endophytic and exophytic features with extensive full thickness dysplasia and atypia with mitotic figures extending to near the surface. Basaloid cells with rare foci suggesting gland formation favoring transitional cell carcinoma.	FGFR3 positive	- Lacrimal sac excision and medial maxillectomy + radiation with 60 Gy in 30 fractions over 4 weeks + chemotherapy with cisplatin + conjunctivo-DCR with a Jones tube - 5 years disease free
Dash et al., 2024 [37]	**Hemangiopericytoma (probably to be recategorized as Solitary fibrous tumor)**	Densely packed spindle cells with a prominent vascular pattern with tumor cells showing irregular shaped nuclei, small nucleoli, and ill-defined cytoplasmic borders.	Not used (STAT6 not assessed)	- Excision of the intact mass in-toto - 3 years disease free
Seethapathi et al., 2021 [38]	**Rhabdomyosarcoma**	Malignant neoplasm comprising small round and spindle-shaped cells scattered in a loose stroma. Large multinucleated bizarre giant cells, atypical mitosis and polygonal cells with eosinophilic cytoplasm.	Not used	- Mass excised in toto and bicanalicular silicone intubation tube in situ + six cycles of chemotherapy - 1 year disease free
Alam et al., 2020 [39]	**Primary apocrine adenocarcinoma**	Focal cribriform pattern of neoplastic cells, duct-like structures, infiltrating desmoplastic stroma with nuclear atypia in the tumor cells, and mitotic activity. Areas of decapitation secretion, highly suggestive of apocrine adenocarcinoma.	CKAE1/AE3, GCDFP15, CK7 positive CK20 negative KI67 was 18–20%.	- Right orbital exenteration + adjuvant RT - 1 year disease free

## Data Availability

The present review was inserted into the Open Science Framework—OSF Public Registry with the registration reference DOI https://doi.org/10.17605/osf.io/hxtsu (Center for Open Science, Charlottesville, VA, USA; the whole protocol can be found at osf.io/37g5a, accessed on 14 September 2025).

## Supplementary Materials

The following supporting information can be downloaded at: https://www.mdpi.com/article/10.3390/cancers17223718/s1, Preferred Reporting Items for Systematic reviews and Meta-Analyses extension for Scoping Reviews (PRISMA-ScR) Checklist.
